# A review of yeast-derived emulsifiers developed through microbial fermentation for the food sector

**DOI:** 10.3389/fmicb.2025.1745931

**Published:** 2026-01-23

**Authors:** Sajad Shokri, Zahrasadat Hashemi, Sona Ayadi Hassan, Christopher J. Chuck

**Affiliations:** 1 Department of Chemical Engineering, University of Bath, Bath, United Kingdom; 2 Department of Biotechnology, Faculty of Biological Sciences, Alzahra University, Tehran, Iran

**Keywords:** biomass, food emulsifier, microbial fermentation, sustainability, yeast

## Abstract

Microbial fermentation is an established technology that is becoming increasingly used to produce key food components. Among the various microorganisms used, yeasts play crucial roles due to their efficiency in synthesizing a wide range of industrially important compounds. The growing demand for sustainable, locally sourced, and animal-free food ingredients has increased the focus on yeast biomass and its derivatives. These yeast-based products, such as food emulsifiers, are a promising next-generation of food components, offering advantages like a low risk of allergenicity. Yeast biomass-based fractions have been effectively used as emulsifiers in various food products including in dairy, meat, bakery, meat alternatives, mayonnaises and salad dressing, with effective properties demonstrated in a range of oil-in-water, water-in-oil, and Pickering emulsion models. Both whole cell biomass and yeast cell fractions such as the yeast cell wall, mannoproteins, glucans, exopolysaccharides and other yeast-derived compounds have been demonstrated to function as effective emulsifiers. An increasingly large number of yeasts, beyond just *Saccharomyces cerevisiae*, have been studied as potential sources of these emulsifiers with the extraction and purification methods employed depending on the specific emulsifier targeted, the required purity, and the intended application. Efficient, cost-effective, and sustainable processes are key to enabling industrial-scale production of these emulsifiers, as such this article reviews the potential yeast-derived food emulsifiers, lists the various yeast species investigated to date, examines the extraction and purification methods, and highlights the potential food applications of these yeast-derived emulsifiers.

## Graphical Abstract

**Figure:**
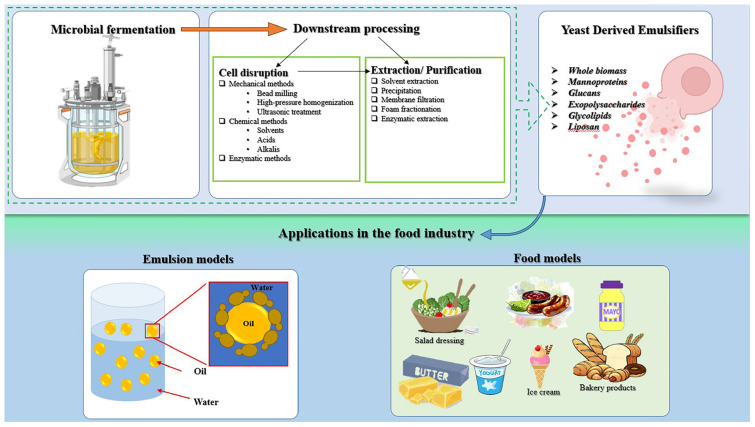

## Introduction

For centuries traditional fermentation has been used to preserve various foods including fish, meat, milk, vegetables, legumes, grains, and fruits. This process relies on the controlled growth of microorganisms, in which organic compounds such as carbohydrates are converted into lower molecule weight species such as organic acids and alcohols (Tofalo and Suzzi, 2016; Maicas, 2023). More recently, microbial cell factories, a refined approach to traditional fermentation, have emerged as a promising strategy for producing high-value industrial compounds for food and pharmaceutical applications (Hilgendorf et al., 2024; Sivadas et al., 2025). In this approach, microorganisms are engineered using methods such as directed evolution, synthetic biology, or advanced genetic engineering to efficiently and specifically produce valuable products. This method allows for the creation of products for use in food, pharmaceuticals, and other industries that are otherwise difficult, costly, or environmentally taxing to produce through traditional methods (Hilgendorf et al., 2024). Among the most common organisms, yeasts-eukaryotic single-celled microorganisms-are preferred as microbial cell factories. Yeasts can grow rapidly, reaching high cell densities, and a number of genetic tools, like CRISPR-Cas9, have been developed (Dudeja et al., 2025). Yeasts are also largely robust, tolerating low pH, high osmotic stress, and toxins, reducing contamination risks. A number of yeast strains have GRAS (Generally Recognized as Safe) status which ensures safety in food and pharma applications, unlike some other microorganisms used across the bioprocessing industry such as *E. coli* (Monteiro et al., 2010; Kulagina et al., 2021; Maicas, 2023). Yeast species such as *Saccharomyces cerevisiae* benefit from a more straightforward regulatory approval process than bacterial species such as *Bacillus subtilis*, even though this bacterium is GRAS and commonly found in fermented foods (Cao et al., 2025). Moreover, in addition to serving as platforms for bioemulsifier production, yeast strains can produce other compounds such as flavoring agents and metabolites with high nutritional value (Johansen et al., 2019; Wang et al., 2025), attributes that are less evident in bacterial systems. Furthermore, yeasts are versatile, able to use a wide range of low-cost feedstocks, including industrial byproducts and food waste, making them a crucial component of sustainable and circular bioeconomy models (Halmos et al., 2018; Kulagina et al., 2021; Maicas, 2023; Wani et al., 2023; Vuc̆urović et al., 2024; Hilgendorf et al., 2024).

The value of yeast biomass yielded from microbial fermentation is significant across food, feed, pharmaceutical, and industrial applications. The yeast biomass, either as whole biomass or yeast cell fractions such as yeast cell wall, mannoproteins, glucans, exopolysaccharides and some other yeast-derived compounds can function as food emulsifiers (Dikit et al., 2010; de Melo et al., 2015; Faustino et al., 2021; Saito et al., 2025; Neto and Silva, 2026). For example, mannoproteins isolated from *Saccharomyces uvarum* has been used as a promising stabilizer and emulsifier in French salad dressing. Interestingly, formulations containing yeast mannoproteins as emulsifier had the highest stability and the highest scores for flavor, color, taste, overall acceptance and purchase intent compared to formulation containing a mixture of mannoproteins and soy lecithin or with soy lecithin alone as emulsifier (de Melo et al., 2015). The improved quality of French salad dressing with yeast mannoproteins is attributed to its amino acid composition. It mainly contains hydrophobic amino acids, followed by neutral, and smaller amounts of hydrophilic amino acids (de Melo et al., 2015). This allows the mannoproteins to bind both apolar and polar regions of different molecules more effectively than soy lecithin. Additionally, the protein part of the mannoproteins provides emulsifying properties by reducing interfacial tension, while the carbohydrate part increases the polymer’s solubility and stabilizes the emulsion (Barriga et al., 1999).

A food emulsifier is a surfactant, a surface-active ingredient that creates a barrier between two immiscible liquids, such as water and oil, so the two can be combined to create stable emulsions [water-in-oil (w/o) and oil-in-water (o/w)]. Emulsifiers are essential in processed foods formulations such as mayonnaise, ice cream, and baked products to create a smooth texture, avoid separation, and extend shelf life (Halmos et al., 2018). While synthetic emulsifiers are widely used due to their specific ionic and structural properties, microbial based emulsifiers offer more chemical diversity, incorporating sugars, amino acids, or fatty acids. This versatility allows them to be used across various food and pharmaceutical industries. Unlike synthetic emulsifiers, biosurfactants produced by microorganisms, are biodegradable and less toxic, posing a lower ecological risk while maintaining similar functional properties. For instance, sophorolipids, a family of glycolipid biosurfactants produced by the non-pathogenic yeast *Candida bombicola*, exhibited relatively lower cytotoxicity on human keratinocytes than chemically synthesized surfactants such as sodium dodecyl sulfate and polyoxyethylene lauryl ether and were classified as “readily” biodegradable chemicals that are degraded 60% within 28 days (Hirata et al., 2009). These features make microbial originated emulsifiers a promising sustainable alternative to synthetic ones (Reis et al., 2023; Vuc̆urović et al., 2024; Roy et al., 2024) i.e., various yeast-derived emulsifiers have been effectively used as emulsifiers in various food products including in dairy, meat, bakery and mayonnaises. Having been demonstrated in different oil-in-water, water-in-oil, and Pickering emulsions (refer to Section “5 Applications of yeast-derived emulsifiers in the food industry”). This review aims to explore the potential of yeast-derived emulsifiers obtained from microbial fermentation for the food sector. It will delve into the relevant aspects of yeast cell structure, detail the different types of bioemulsifiers obtainable from both whole yeast biomass and its fractions, cover the methods for their extraction and purification, discuss their key functional properties, and highlight their diverse applications in various food products.

## 2 Yeast cell structure

**FIGURE 1:**
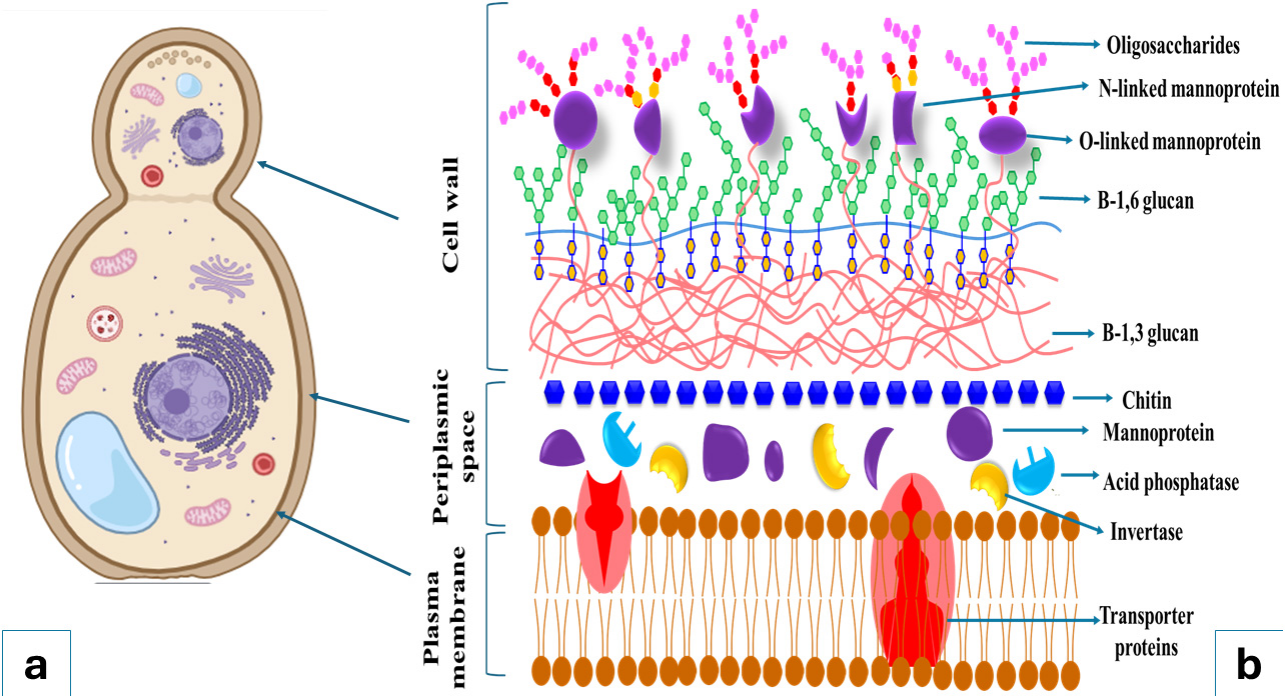
Yeast cell main components **(a)** and its cell wall and membrane structures **(b)**.

**FIGURE 2:**
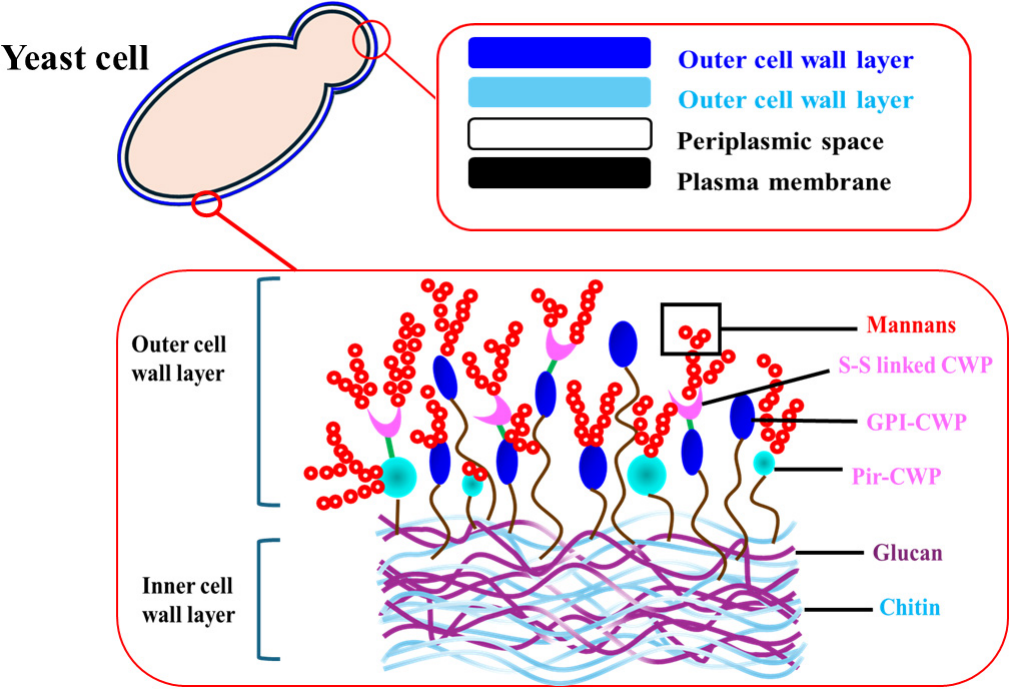
Typical yeast cell wall outer and inner layers.

Yeast cells have membrane-bound organelles and other ultrastructural characteristics common to other eukaryotic cells (Figure 1b). The term “yeast” and “*Saccharomyces cerevisiae*” are frequently, albeit incorrectly, used interchangeably. Depending on the species and conditions of growth, the size of yeast cells can vary widely; for example, while *Saccharomyces cerevisiae* cells are typically 5–10 μm in diameter, some *Candida* species can reach lengths of 20–50 μm. In general, yeast cell width is less variable, ranging from approximately 1 to 10 μm (Tofalo and Suzzi, 2016). Yeast cell wall is a dynamic structure, undergoing constant remodeling to adapt to various environmental stresses and cell growth (Narsipur et al., 2024). For example, the percentage of protein, neutral carbohydrates, chitin, alkali-soluble fraction, alkali-insoluble fraction, and monosaccharide composition (glucose + mannose) in *Saccharomyces cerevisiae* cell wall are 13.5, 85, 3.36, 33.5, 37.3, and 80–90 + 10–20%, respectively, whereas these components in *Yarrowia lipolytica* cell wall are 15, 70, 7, 38.8, 61.2, and 60 + 40% (Khot et al., 2020). However, while considerable information is available on the cell wall structure of *Saccharomyces cerevisiae*, there is limited knowledge in the literature regarding the cell wall composition of other important yeast species used as a source of food emulsifiers. The *Saccharomyces cerevisiae* cell wall is composed of macromolecules, including two distinct forms of β-glucans [β-(1–3)- D-glucan (25%) - β-(1–3)-D-glucan bounded to chitin (35%) – and β-(1–6)-D glucan (5%)], chitin (1%–2%), and highly glycosylated glycoproteins, commonly referred to as mannoproteins (35%) (Faustino et al., 2021). A schematic representation of *S. cerevisiae* cell wall is shown in Figures 1a, 2. The cell wall typically consists of two layers (inner and external) accounts for approximately 15%–30% of the cell’s total dry mass (Faustino et al., 2021; Bastos et al., 2022). The inner layer contains β-1,3-glucan (50%–55% of the cell wall dry weight) and chitin (1%–2%), and the external layer contains mainly mannoproteins (35%–40%) and β-1,6-glucan (Figure 1a; Gonzalez-Ramos et al., 2008). Glucans and mannoproteins are the two main components of the yeast cell wall.

### 2.1 Glucans

Glucans contribute to the cross-linking of cell wall components and maintain its structural integrity. Due to their chemical and structural characteristics, they have been considered as thickening and emulsifying agents in the food industry (Faustino et al., 2021; Łukaszewicz et al., 2024). Water-soluble, alkali-soluble, and alkali-insoluble glucans are three classes of *Saccharomyces cerevisiae* cell wall glucans (Bastos et al., 2022). The majority are alkali-insoluble (β1→3)-D-glucans that can be linked to Glc residues and chitin chains. In general, cell wall β-glucans in yeast species are composed of (β1→3)- and (β1→6)-D-glucose linkages. This structural organization has been described in several yeasts, including *Aureobasidium pullulans* (Han et al., 2020), *Candida albicans* (Bekirian et al., 2024), and *Saccharomyces* spp. (Bastos et al., 2022). Predominantly, (β1→3)-D-glucans branched via (β1→6)-D-Glc bonds constitute approximately 80%–90% of the inner cell wall layer, accounting for up to ∼50% of the total cell wall, and form a dense fibrous network. The (β1→3)-glucans function as covalent attachment site to the other yeast cell wall components (Bastos et al., 2022). (β1→6)-D-glucans are highly branched polysaccharides that are shorter than (β1→3)-D-glucans constitute 8%–18% of the cell wall inner layer (and up to 10% of yeast cell wall) and playing an adhesive role in cell wall organization (Bastos et al., 2022). Yeast β-glucan is a key polysaccharide with thickening, water retention, oil-binding, and emulsifying properties with a promising potential in the food industry for its ability to stabilize emulsions and enhance texture of food products (Zeko-Pivac̆ et al., 2023; Łukaszewicz et al., 2024). For example, β-glucan derived from brewer’s spent yeast byproduct have demonstrated superior stabilizing properties compared to commercially available alternatives when used to replace xanthan gum in mayonnaise formulation (Zeko-Pivac̆ et al., 2023).

### 2.2 Mannoproteins

Mannoproteins, found on the yeast cell’s outer surface, are the second most abundant component of the yeast cell wall and have demonstrated unique emulsifying and stabilizing properties in food formulations (da Silva Araújo et al., 2014; Timira et al., 2024). They are bound to the inner layer and make up 40% of the yeast cell wall (dry weight) (Yammine et al., 2022). The mannoproteins can be bound to the polysaccharide layer by covalent or non-covalent bonds (Yammine et al., 2022). The covalently bound mannoproteins, known as cell wall proteins (CWPs) can be categorized into three major groups based on their molecular linkage type: GPI-CWP group including most of CWPs linked through a glycosylphosphatidylinositol (GPI) remnant, PIR-CWP group comprising CWPs linked through an alkali sensitive bond, and the group of proteins linked by disulfide bridges to other CWPs (Yammine et al., 2022). Depending on the structure of the cell wall, mannoproteins can attach to either β-(1,3)-glucan or β-(1,6)-glucan using a GPI anchor (Timira et al., 2024). The GPI anchor is an important mechanism that helps select proteins with a C-terminal signal sequence and is used to bind these proteins to the plasma membrane and cell wall (Timira et al., 2024). Mannoproteins are highly glycosylated polypeptides, containing 50%–95% mannans (Yammine et al., 2022). In *Saccharomyces* species, cell wall mannoproteins are classified into N-linked and O-linked types. N-linked mannoproteins consist of 90% carbohydrate and 10% protein, whereas O-linked mannoproteins have a higher protein content of 50%. The number of α-linked mannose units differs between the two types; N-linked mannoproteins have between 50 and 200 units, whereas O-linked mannoproteins have only up to 5 units. Even though O-linked chains are short, many cell wall proteins have serine- and threonine-rich regions, allowing for a large number of O-linked chains per protein. As a result, the yeast cell wall contains a significant amount of O-linked mannose. Additionally, yeast mannoproteins contain phosphorylated mannose residues, which introduce negative charges to the cell wall and play a role in yeast water retention, flocculation, and protection (Lesage and Bussey, 2006; Loibl and Strahl, 2013; Bastos et al., 2022). *Saccharomyces boulardii* produces mannoproteins with a high mannose-to-glucose ratio, which contribute to functional properties such as improving wine quality and enhancing protein and tartrate stability. The molecular weights of these mannoproteins range from 50 to 500 kDa, and their glycan moieties are composed predominantly of mannose (Snyman et al., 2023a). In contrast, mannoproteins from *Metschnikowia fructicola* exhibit much higher molecular weights (approximately 1300 kDa) and display distinct saccharide-to-protein ratios, resulting in structural differences that influence their interactions with other molecules (Snyman et al., 2023b). Yeast mannoproteins have shown a promising potential as effective food emulsifiers due to their unique structural and functional properties. They possess amphiphilic characteristics that enable them to stabilize oil-in-water emulsions. Their natural origin, biocompatibility, and ability to improve the texture, stability, and shelf life of food products make them an appealing alternative to synthetic emulsifiers.

The emulsifying properties of yeast mannoproteins is due to the combined roles of their carbohydrate and protein moieties. The carbohydrate portion enhances water solubility, provides steric stabilization, and may contribute to electrostatic repulsion, preventing droplet coalescence (Li and Karboune, 2019; Timira et al., 2024). Meanwhile, the protein component binds to the oil-water interface through hydrophobic interactions, reducing interfacial tension and stabilizing emulsions. This synergy between the hydrophilic carbohydrate and amphiphilic protein moieties enables yeast mannoproteins to act as effective natural emulsifiers in food systems (Qiao et al., 2022). Emulsifying activity of mannoproteins generally decline when the pH of the extract increases. However, mannoproteins extracts with complex protein and glycosylated component features have less pH-dependent emulsion stability (Timira et al., 2024). The stability of emulsions in which yeast mannoproteins is used as an emulsifier can also be influenced by salts; for instance, adding CaCl_2_ can reduce droplet electrostatic repulsion, leading to emulsion destabilization (Timira et al., 2024).

Beyond the core structural components discussed above, yeast cells contain various other constituents that contribute to their overall physiology. Chitin, which helps maintain cell rigidity and morphology, is a linear polymer of 1–4 linked N-acetylglucosamine units (Faustino et al., 2021). Yeast’s surface plasma membrane is a lipid bilayer that contains enzymes for cell wall formation, signal transduction, and transport, as well as proteins that function as a cytoskeleton (Figures 1a, b; Tofalo and Suzzi, 2016). Phospholipids and sterols (primarily ergosterol and zymosterol) comprise the majority of lipid components in yeast cell structure (Tofalo and Suzzi, 2016). Yeast-derived glycolipids, recovered from the cell membrane lipid extracts or from fermentation broth, can be used as flavors in food products as emulsifiers and surfactants (Tofalo and Suzzi, 2016). The periplasmic space, located 35–45 Å outside the plasma membrane, contains proteins such as phosphatase and invertase that cannot pass through the cell wall (Tofalo and Suzzi, 2016). However, the structural composition of yeast cells can vary significantly depending on various factors such as the growth conditions, the point in the life cycle, species, and strain (Tofalo and Suzzi, 2016). To date, many of these biomaterials have been used as emulsifiers and stabilizers in the food industry (Pavlova et al., 2011; Narsipur et al., 2024).

## 3 Biosurfactants and bioemulsifiers derived from yeast

Microbial surfactants are amphiphilic, surface-active compounds that reduce surface and interfacial tension by accumulating at the interface between two immiscible phases. Their amphiphilic structure consists of a non-polar hydrocarbon chain and a polar moiety that may be ionic, non-ionic, or amphoteric (de Fernandes et al., 2023). A key parameter determining biosurfactant efficiency is the critical micelle concentration (CMC), defined as the minimum concentration at which biosurfactant molecules self-assemble into micelles in solution (Souza et al., 2014; Ramesh and Sakthishobana, 2021). Above the CMC, micelle formation facilitates emulsion development and enables the dispersion of hydrophobic compounds in aqueous systems and vice versa (Souza et al., 2014; Santos et al., 2016).

Many microbial biosurfactants exhibit low CMC values, meaning that smaller quantities are required to achieve effective emulsification compared to synthetic surfactants (Santos et al., 2016; Hashemi et al., 2024). Yeast-derived biosurfactants illustrate this efficiency well; for example, *Pichia pseudolambica* whole-cell biosurfactants show CMC values ranging from 7 to 16 mg/mL (Macedo Silva et al., 2024). Similarly, CMC values of 43.63 and 24 mg/L have been reported for biosurfactants produced by *Cyberlindnera fabianii* and a newly identified yeast species (JAF-11) isolated from the flower *Prunus mume* Sieb. *et* Zucc., respectively (Kim et al., 2023; Eryasar-Orer et al., 2025).

Microbial biosurfactants are commonly classified based on microbial origin, chemical structure, molecular weight, and cellular localization (Varjani and Upasani, 2017; Valkenburg et al., 2024). Low-molecular-weight compounds, such as glycolipid biosurfactants including sophorolipids and mannosylerythritol lipids (MELs), are typically referred to as biosurfactants. In contrast, high-molecular-weight polymeric compounds, such as liposan and mannoproteins, are classified as bioemulsifiers (Varjani and Upasani, 2017; Valkenburg et al., 2024). While biosurfactants primarily reduce surface and interfacial tension, bioemulsifiers are particularly effective in stabilizing oil-in-water emulsions. Although these terms are sometimes distinguished in the literature, they are used interchangeably in this review.

Yeast-derived biosurfactants are of particular interest for food applications due to their low toxicity, high biodegradability, and functional stability across a wide range of pH, temperature, and salinity conditions (Pardhi et al., 2022; Sarubbo et al., 2022). Most biosurfactant-producing yeasts are non-pathogenic and non-opportunistic, making them suitable candidates for food-related uses. In addition, some yeast species can produce higher biosurfactant yields than bacterial counterparts (de Fernandes et al., 2023). *Saccharomyces cerevisiae*, a GRAS-designated yeast, is a well-studied biosurfactant producer with promising applications in the food industry (Roy et al., 2024). Recently, three recombinant cell-wall-associated proteins from *S. cerevisiae*–fructose-1,6-bisphosphate aldolase 1, enolase 1 and 2, and triose-phosphate dehydrogenase 2–were shown to exhibit strong emulsifying activity (Saito et al., 2025). Due to their relatively small size, lower glycosylation levels, and functional performance, these proteins represent promising alternatives to conventional food emulsifiers.

Several yeast genera, including *Candida*, *Rhodotorula*, *Saccharomyces*, and *Wickerhamomyces*, are recognized producers of biosurfactants. Notably, high biosurfactant yields (up to 120 g/L) have been reported for *Candida bombicola* cultivated on waste substrates from the meat processing industry, highlighting the potential for sustainable large-scale production (de Fernandes et al., 2023).

### 3.1 Glycolipids

Glycolipids are the most common and well-studied type of biosurfactants. They are a diverse group that consist of a sugar unit such as glucose, rhamnose, mannose, or galactose linked to a hydrophobic part such as a fatty acid, fatty alcohol, or hydroxy fatty acid (Banat et al., 2014; Varjani and Upasani, 2017). Examples of glycolipids include rhamnolipids, sophorolipids, and trehalolipids (Azevedo et al., 2024). Rhamnolipids are the most studied and well-known microbial-derived biosurfactants, valued for their unique properties and potential applications in the food industry. Rhamnolipids are glycolipids comprised of one or two α-l-rhamnose units, mainly linked to one or two 3-hydroxyl fatty acid moieties. Rhamnolipids are typically produced in a variety of structural variations with differing chain lengths of hydroxyl fatty acids (Henkel et al., 2017). *Pseudomonas aeruginosa*, a Gram-negative bacterium, is the primary producer of rhamnolipids, and their biosynthesis has been extensively studied due to their role in virulence (Sarubbo et al., 2022). However, as an opportunistic human pathogen, *P. aeruginosa* is not ideal for industrial-scale production, despite the safety of the purified product (Henkel et al., 2012). Recently, a commercial rhamnolipid fermentation facility was established by Evonik, demonstrating industrial feasibility (Wongsirichot and Winterburn, 2025). To address biosafety concerns, rhamnolipid production has been engineered in the GRAS yeast *Saccharomyces cerevisiae*, enabling the use of inexpensive substrates such as sucrose (Bahia et al., 2018). Nevertheless, reduced growth rates following genetic modification and limitations in precursor availability (e.g., dTDP-L-rhamnose), carbon flux optimization, and expression systems remain key bottlenecks for improving yields in yeast hosts (Wittgens and Rosenau, 2020). Notably, rhamnolipids produced by *S. cerevisiae* URM 6670 have been successfully applied as food emulsifiers, with patented uses in bakery and confectionery products (de Fernandes et al., 2023; Azevedo et al., 2024). It also can be utilized as nanocarriers, nanoemulsions, lipid-based nanosystems and nanostructures in the food industry (Santos et al., 2016; Azevedo et al., 2024).

Similar to rhamnolipids, Sophorolipids are among the most well-known microbial glycolipids. *Candida* sp., *Rhodotorulla* sp., *Wickerhamiella* sp., and *Yarrowia* sp. are some of the well-known yeast species sources for sophorolipids production. The carbohydrate part of sophorolipids is a glucose-based oligosaccharide with a unique β-1,2 glycosidic bond, which may or may not be acetylated at the 6′ and/or 6′′ ends. This is linked to a fatty acid chain that is 14–18 carbon atoms long. The fatty acid chain either has a carboxyl group at one end or is esterified internally, typically at the 4′ position, and sometimes at the 6′ and/or 6′′ positions, forming a lactonic fatty acid. Sophorolipids properties such as being highly biodegradable, low in toxicity, having anti-adhesive, antimicrobial, and emulsifying properties, making them promising candidates for use as food emulsifiers as well as food preservatives in the food industry (Gaur et al., 2019; de Fernandes et al., 2023; Purohit et al., 2024). Mannosylerythritol lipids (MELs) are another example of a microbial originated glycolipid biosurfactant. Four natural variants of MELs (A, B, C, and D) have been identified, all featuring a hydrophilic core made of 4-O-β-D-mannopyranosyl-D-erythritol. These variants differ in their acylation at the C4 and C6 positions of the mannose. Variant A is diacylated, variants B and C are monoacylated at C4 and C6, respectively, and variant D is deacylated. Hydrophobic fatty acid chains are attached to the sugar core, with di-acetylated MELs being the most common. The production of different MEL variants and the degree of fatty acid saturation depend on the microorganism and carbon source used (Valkenburg et al., 2024). MELs are produced by a variety of basidiomycetous yeasts mainly belonging to the genera *Pseudozyma* and *Candida* (Valkenburg et al., 2024).

### 3.2 Liposan

Liposan is another yeast-originated bioemulsifier produced extracellularly by *Yarrowia lipolytica*, consisting of 83% carbohydrates and 17% proteins, with no fatty acids in its structure, making them water-soluble (Santos et al., 2016). Liposan is widely utilized in the food and cosmetic industries to create stable oil-in-water emulsions (Pincbuk, 2000; Cirigliano and Carman, 1985; Santos et al., 2016). Mannoproteins, which constitute 35%–40% of the yeast cell wall, also exhibit excellent emulsifying and stabilizing properties in various food formulations (detailed in Section “4 Extraction methods of yeast originated emulsifiers”) (Li et al., 2020).

### 3.3 Whole biomass

*Saccharomyces cerevisiae* biomass is composed mainly of proteins (up to 49% DW) and carbohydrates (up to 54% DW) (Łukaszewicz et al., 2024). The potential of *S. cerevisiae* whole-cell to stabilize various emulsions and droplets has been shown (Narsipur et al., 2024). A study showed stabilizing ability of *S. cerevisiae* yeast in oil-in-water (O/W) emulsions. The washed yeast cells showed that intracellular material secreted or cell fragments of yeast provide more stable emulsions (Moreira et al., 2016). In another study inactivated baker’s yeast, *S. cerevisiae*, was used to generate and stabilize O/W emulsion models. The yeast cells acted as Pickering-type stabilizers by residing at the oil–water interface. The contact angle of the yeast at the oil–water interface demonstrated its ability to stabilize O/W emulsions. These yeast cells may be used in the design of processed food emulsions as well as for the replacement of common synthetic surfactants (Firoozmand and Rousseau, 2016). Firoozmand and Rousseau (2022) used *S. cerevisiae* cells as emulsification agent to prepare O/W emulsions using 7%, 8%, and 9% yeast biomass, 60%, 65%, and 70% oil, and 23%, 27%, and 31% water. Yeast cells stabilized O/W emulsions by settling at the oil-water interface; in emulsion formulations with less oil content a higher yeast percentage was required to create more oil droplets and increase the total oil phase volume (Firoozmand and Rousseau, 2022). Both yeast cell adhesion and dispersed cell materials play remarkable roles in emulsion stabilization (Moreira et al., 2016); fewer whole cells and more cell material likely compete with yeasts at the droplet interface, enhancing emulsion stabilization (Dickinson, 1992; Moreira et al., 2016). Whole *S. cerevisiae* yeast from both bakery and brewing sources contains substantial amounts of protein (41.7%–44.6%, w/w), glucan (19.2%–22.7%, w/w), and mannan (12.7%–14.4%, w/w). These components, individually and collectively, demonstrate significant potential as emulsifying agents. The recovery of yeast extract from brewer’s yeast biomass is 65.3% (w/w), with a high mannan-to-protein ratio in its mannoprotein fraction. This high mannan-to-protein ratio is a crucial factor for the efficacy of yeast biomass-based emulsifiers (Li et al., 2020). In addition, *S. cerevisiae* is an efficient tool for the production of protein biomass for various purposes including being considered as food emulsifier (Narsipur et al., 2024; Timira et al., 2024). The potential of protein fractions of yeast biomass have also been shown to stabilize emulsions in various food products such as dressings, muffins, sauces, and mayonnaise (Timira et al., 2024). In addition to *S. cerevisiae*, other yeast species such as *Saccharomyces boulardii*, *Candida albicans*, and *Kluyveromyces marxianus* can serve as effective sources of protein biomass to stabilize emulsions in the food manufacturing (Narsipur et al., 2024). *Yarrowia lipolytica*, with a protein content of 54%–77%, also compares favorably to *S. cerevisiae* (50%–60%) as an approved protein-rich biomass source (Timira et al., 2024).

### 3.4 Exopolysaccharides

Yeast-derived exopolysaccharides (EPS) include linear mannans, pullulan, glyco-oligosaccharides, galacto-oligosaccharides, and other heteropolysaccharides (Gientka et al., 2015). The antarctic yeast *Cryptococcus laurentii* AL100 produces a new EPS containing arabinose, mannose, glucose, galactose and rhamnose with emulsifying and stabilizing properties in O/W emulsions (Pavlova et al., 2011). *Saccharomyces cerevisiae* Y3 exopolysaccharides contained 83.65% of total sugars, uronic acid, protein and sulfuric acid groups has shown good emulsifying ability (Liu et al., 2022). *Sporidiobolus pararoseus* PFY-Z1 exopolysaccharides, mainly composed of mannose followed by glucose, are suitable as an emulsifier in the food industry (Liu et al., 2023; Xue et al., 2023). EPS produced by *Rhodobacter johrii* CDR-SL 7 Cii and *Rhizobium radiobacter* CAS showed emulsion stabilization activity in some food applications (Thraeib et al., 2022). In another study, using a galactan EPS from *Weissella confusa* KR78067, food-grade flavor emulsions, specifically vanilla and cardamom were evaluated. The emulsions exhibited desirable physical properties, smooth spherical appearance, and improved sensory characteristics when incorporated into muffins, demonstrating galactan EPS as a promising bioemulsifier for flavor and bioactive compound delivery in food and pharmaceutical applications (Kavitake et al., 2020).

### 3.5 Other extra and/or intracellular yeast originated potential food emulsifiers

*Yarrowia lipolytica* produces considerable amounts of phospholipids with emulsification properties (Szczepańska et al., 2022). *Saccharomyces uvarum* is another yeast with potential of producing emulsifying compounds including phospholipids (Hamza et al., 1994). Some of the yeast candidates as sources for the bioproduction of food emulsifiers are listed in Table 1.

**TABLE 1:** Yeast candidates for bioproduction of food emulsifiers – biosurfactants produced and their functions.

Yeats genus/ species	Bio-emulsifier (feature/name)	Function/application	References
***Saccharomyces***			
*Saccharomyces cerevisiae*	Mannoproteins	Emulsification, stability, and foaming capabilities, replace synthetic emulsifiers and stabilizers in food, improve gut health and immunological function	Timira et al., 2024
*Saccharomyces cerevisiae* URM 6670	Glycolipid (high percentage of linoleic acid)	Thermal stability (up to 200°C), completely replaces egg yolk (4%) in a cookie dough formulation.	Ribeiro et al., 2020
*Saccharomyces cerevisiae* Y3	Exopolysaccharides containing sugars, uronic acid, protein and sulfuric acid groups	Good emulsifying ability (more than 25%), applicable in the food industry, especially in the dairy industry as a thickener, stabilizer, and emulsifier.	Liu et al., 2022
*Saccharomyces cerevisiae* KA01	Mannoproteins	Emulsion activity (65%), stabilize oil-in-water emulsions at pH 5–8 and extreme temperature and salinity, potential applications in salad dressing	Dikit et al., 2010
*Saccharomyces uvarum*	Mannoproteins	Increased stability during the refrigerated storage time, good emulsifying and stabilizing property, to replace xanthan gum in mayonnaise formulation.	da Silva Araújo et al., 2014
***Candida***			
*Candida lipolytica*	Liposan, inducible water-soluble bioemulsifier	A reduction in emulsion decay in the presence of 0.8 mg liposan, emulsify and stabilize (vegetable) oil in water emulsions.	Cirigliano and Carman, 1985
*Candida (Torulopsis) apicola*	Glycolipid, sophorose lipid	Considered as bioemulsifier with potential application in the food industry.	Thraeib et al., 2022; Hommel et al., 1994
*Candida utilis*	Carbohydrate–lipid–protein complex, produced in a medium containing waste frying canola oil	Improved consistency with 0.7% (w/v) biosurfactant and guar gum, incorporation in salad dressing formulation.	Campos et al., 2019
*Candida bombicola* URM 3718	Biosurfactant/bioemulsifer, produced in a medium containing sugarcane molasses, residual soybean oil and corn steep liquor	Increasing the stability of the emulsions, incorporated into the cupcake formulation as a substitute for vegetable fat, stable at 180 °C.	Rubio-Ribeaux et al., 2023; Silva et al., 2020
***Yarrowia***			
*Yarrowia lipolytica*	Yansan, lipid–carbohydrate–protein complex	High emulsification activity and stability in pH range of 3–9, stabilize water-in-oil and oil-in-water emulsions, potential application in food.	Amaral et al., 2006
***Kluyveromyces***			
*Kluyveromyces marxianus*	Mannan	Suggesting mannan as a safe and effective bioemulsifier for using in food products.	Hajhosseini et al., 2020
*Kluyveromyces marxianus*	Mannoprotein, anionic polysaccharide	Effectively stabilize oil-in- water emulsions in a range of pH, ion and temperatures.	Hajhosseini et al., 2021
***Sporidiobolus***			
*Sporidiobolus pararoseus* PFY-Z1	SPZ, exopolysaccharide	Emulsifying ability, used as a thickener, stabilizer and emulsifier in the food, potential functional properties for human health.	Xue et al., 2023
***Rhodococcus***			
*Rhodococcus qingshengii* QDR4-2	QEPS, containing mannose and glucose	Excellent emulsifying capabilities (72.75%), potential as a bioemulsifier in food.	Li et al., 2023
***Wickerhamomyces anomalus***			
*Wickerhamomyces anomalus* PY189	Glycolipid biosurfactant	Forming stable oil-in-water emulsion, applied to the encapsulation of lemongrass oil for food industry.	Dejwatthanakomol et al., 2016

## 4 Extraction methods of yeast originated emulsifiers

The extraction of yeast originated emulsifiers involves a series of methods designed to release and purify the target compounds while maintaining their functional integrity, each method has its strengths and limitations. Mechanical, chemical, and enzymatic methods offer different balances of efficiency and cost, while precipitation, solvent extraction, and membrane filtration provide avenues for purifying the extracted emulsifiers. Although the choice of method depends on the specific emulsifier targeted, the required purity, and the intended application, it is crucial to identify efficient and cost-effective processes for the extraction and purification of yeast originated emulsifiers, given that over 60%–80% of the costs associated with the production are attributed to downstream processing (Najmi et al., 2018). Advances in integrating these methods and developing more sustainable processes continue to enhance the feasibility of industrial-scale production of yeast-derived emulsifiers. Below is an overview of the most common and effective extraction methods used to obtain yeast emulsifiers, where Table 2 briefly summarizes each technology.

**TABLE 2:** The most common and effective extraction methods used to obtain yeast emulsifiers.

**Method**	**Advantages**	**Disadvantages**
Cell disruption	-Effective for large-scale production	-Non-selective -Co-extraction of unwanted cellular components
Solvent extraction	-High yields -Straightforward protocols -Obtaining pure emulsifiers -Allowing for scalability in industrial applications	-Selective isolation of specific emulsifiers remain challenging -Costly -Toxic waste -Complicated
Precipitation	-Simplicity -Cost-effectiveness - Minimal equipment -Easy to implement -Suitable for both lab-scale and industrial-scale operations	-Co-precipitation of other biomass residues, additional purification steps - Acid handling and disposal
Membrane filtration	-Non-toxic -Scalable -Environmentally friendly -Less energy -Highly efficient -High purity -High recovery	-Membrane fouling -Reduced process efficiency -Increased cleaning costs -Equipment downtime -Not highly selective -Collecting unwanted small molecules
Foam fractionation	-Promising method for large-scale applications of biosurfactants -Cost-effective -Minimal energy input -Continuous emulsifier recovery -Eco-friendly	-Effectiveness depends on the foaming properties of the biosurfactant -Unintentionally removed biomass along with the foam -Reducing reactor productivity -Challenges in maintaining consistent foam stability -Need for specialized equipment
Enzymatic extraction	-Selective and eco-friendly -Highly selective -Preserves the functional structures -Mild -Environmentally sustainable	-Expensive -Time-consuming -Require optimization
Crystallization	-Key purification method for certain emulsifiers	-Time-consuming -Demands careful control
Combining methods	-Optimized yield -Maintain the integrity -Balancing efficiency -Cost-effectiveness -Improve the separation -Improve emulsifier yields	

### 4.1 Cell disruption methods

Cell disruption methods are commonly used for breaking down yeast cells to release intracellular emulsifiers. These methods are effective for large-scale production but come with the downside of being non-selective, often leading to the co-extraction of unwanted cellular components (Gautério et al., 2023). To extract valuable compounds from yeast cell biomass, various cell disruption methods are employed. Mechanical methods include bead milling, high-pressure homogenization, and ultrasonic treatment, all of which use physical forces to break down cell walls have all been reported. Chemical methods involve the use of solvents, acids, and alkalis to weaken cell walls, facilitating the release of intracellular materials, though they may introduce contaminants. Enzymatic methods utilize specific enzymes like glucanases and proteases to selectively degrade cell wall components, preserving the functionality of the extracted compounds. Emerging techniques such as microwave-assisted extraction, pulsed electric fields, and high voltage electrical discharge leverage non-thermal processes to disrupt cells efficiently. Combining these methods can optimize yield and maintain the integrity of the extracted compounds, balancing efficiency with cost-effectiveness (Liu et al., 2016; Gautério et al., 2023).

### 4.2 Solvent extraction

Solvent extraction is one of the most common and efficient methods for recovering yeast emulsifiers such as mannosylerythritol lipids (MELs), rhamnolipids, and mannoproteins, with high yields and relatively straightforward protocols (Vélez-Erazo et al., 2022; Venkataraman et al., 2022; Valkenburg et al., 2024). This method takes advantage of emulsifiers’ solubility in organic solvents. A typical protocol involves mixing an equal volume of solvent with the mixture containing the yeast emulsifier. After shaking, the organic layer is separated and evaporated to produce an emulsifier-rich concentrate. A variety of polar and non-polar solvents are used based on the specific emulsifier being targeted. The most commonly used solvents include ethyl acetate, chloroform, methanol, and butanol. Ethyl acetate is commonly used for sophorolipids (Hubert et al., 2012), while a chloroform–methanol mixture (2:1 v/v) is preferred for rhamnolipids (Joy et al., 2020). In MEL production, ethyl acetate is widely used, and studies report a recovery rate of up to 90% when MELs are extracted from *Pseudozyma aphidi* yeast (Rau et al., 2005). Solvent extraction is particularly useful in the laboratory for obtaining pure emulsifiers and allows for scalability in industrial applications. While solvent extraction is practical for recovering glycolipids and lipopeptides, selective isolation of specific emulsifiers remain challenging due to their physical similarities (Rangarajan and Clarke, 2016). The process can also be costly, given the high solvent consumption. In many cases, toxic waste is generated, which requires proper disposal, increasing operational costs. Furthermore, solvent recovery is complicated by the formation of stable azeotropes (Nascimento et al., 2023). Recent advancements have aimed to integrate solvent extraction with other extraction and purification methods, such as precipitation and coagulation, to reduce solvent usage. This combined approach minimizes environmental impact and increases recovery efficiency. For example, the synergistic effect of sweep floc coagulation with 0.4% (w/v) FeCl_3_, followed by acidification to pH 2.0 (using 6 N HCl) and solvent extraction with chloroform: methanol (2:1 v/v), resulted in a maximum rhamnolipid recovery of 97.5% from fermentation broth, compared to 89.05% recovery with simple acidification followed by solvent extraction (Joy et al., 2020).

### 4.3 Precipitation

Precipitation is a widely used technique for recovering emulsifiers after cell disruption. Acid precipitation is particularly popular for extracting emulsifiers due to its simplicity, cost-effectiveness, and ability to purify emulsifiers with minimal equipment (Vélez-Erazo et al., 2022). In this method, strong acids such as hydrochloric acid (HCl) are added to the mixture containing yeast emulsifiers to lower the pH (typically to around 2.0–3.0). When the pH drops, emulsifiers become positively charged, leading to flocculation. As their weight increases, they precipitate due to van der Waals forces, attracting each other (Baker and Chen, 2010). The solution is then cooled (often overnight), and centrifugation is used to collect the precipitated emulsifier (Venkataraman et al., 2022). Acid precipitation is effective for emulsifiers like glycolipids and lipopeptides, particularly those from *Pseudozyma* species (Coutte et al., 2017). For example, the acid precipitation of MELs at pH 3 was successful in separating emulsifiers from residual oils and proteins in the fermentation broth. Acid precipitation has been shown to yield significant results in the extraction of emulsifiers from *Pseudozyma antarctica*, where a pH reduction led to the precipitation of MELs, which were then recovered using solid-liquid separation techniques (Kitamoto et al., 1990a). Acid precipitation method is low-cost, easy to implement, and does not require sophisticated equipment, making it suitable for both lab-scale and industrial-scale operations. It is particularly effective when paired with subsequent purification steps like solvent (n-hexane) extraction (de Fernandes et al., 2023). The downside to acid precipitation is the co-precipitation of other biomass residues such as proteins and oils (Kitamoto et al., 1990b), which may require additional purification steps to ensure high emulsifier purity. The need for acid handling and disposal can also complicate the process at an industrial scale. Ammonium sulfate precipitation is another approach used to target high-molecular-weight emulsifiers via converting amphiphilic compounds such as glycolipids to hydrophobic compounds. By adjusting the saturation of ammonium sulfate, specific proteins and polysaccharides can be selectively precipitated, which are then further purified through dialysis or other methods such as lyophilises. This technique is effective but may involve multiple steps, increasing the complexity of the process (Venkataraman et al., 2022; de Fernandes et al., 2023). Chioru et al. (2024) reported a cost-effective alkaline–acid extraction method for extracting β-glucans from wine lees, using NaOH followed by acetic acid or HCl to disrupt the yeast cell wall and solubilise the yeast β-glucan. Similarly, Mahmoud Amer et al. (2021) demonstrated enhanced biological activity of yeast β-glucans extracted using a modified NaOH–acetic acid method.

### 4.4 Membrane filtration

Membrane filtration, including ultrafiltration, is a popular non-toxic and scalable method used for the extraction of emulsifiers. This technique relies on molecular size differences to separate emulsifiers from other compounds in a mixture containing emulsifiers. Ultrafiltration involves passing the solution through membranes such as polysulfone and polyethersulfone membranes and regenerated cellulose membranes with specific pore sizes that retain emulsifiers and allow smaller molecules to pass through (Jauregi and Kourmentza, 2019). Ultrafiltration is commonly employed for biosurfactants such as MELs and rhamnolipids. A study using 100 kDa MWCO ultrafiltration membranes recovered about 80% of MELs from *Pseudomonas tsukubaensis* cultured in cassava wastewater. When scaled up from 20 to 500 mL using a crossflow filtration unit, the results remained consistent, maintaining the same recovery rate (de Andrade et al., 2017). Ceramic membranes are being explored for industrial-scale applications due to their higher resistance to solvents and extended operational lifespan compared to polymeric membranes (de Andrade et al., 2022).

Membrane filtration is relatively easy to scale up, and it does not generate harmful chemical residues, making it ideal for the food industry. The process consumes less energy than traditional solvent extraction and is highly efficient in recovering emulsifiers with a high purity and a high recovery rate (Venkataraman et al., 2022). Nevertheless, a major challenge of membrane filtration is membrane fouling, which can reduce process efficiency over time (Valkenburg et al., 2024). Fouling can lead to increased cleaning costs and equipment downtime. Additionally, membrane filtration is not highly selective, as it separates compounds based solely on size, potentially collecting unwanted small molecules along with the emulsifiers (Jauregi and Kourmentza, 2019). Researchers are exploring combining membrane filtration with solvent extraction to improve the separation of emulsifiers from other small molecules. For example, a study by Nascimento et al. (2023) developed a downstream process to separate molecules with similar molar masses, particularly triacylglycerols from MEL. This study investigated a method for microbial biosurfactant production that initially removes 90% of triacylglycerols and subsequently separates other lipid derivatives via organic solvent nanofiltration. The performance of three commercial membranes (GMT-oNF-2, PuraMEm-600, and DuramMem-500) and several polybenzimidazole (PBI) membranes, fabricated with 22%, 24%, or 26% PBI solutions, was evaluated. It was observed that achieving higher purities through increased diavolumes correlates with increased product losses. However, a two-stage sequential cascade filtration utilizing the GMT-oNF membrane resulted in 98% purity of reagent-grade MELs (≥97% purity) with a 11.6% loss of MELs. Decoloration was effectively achieved using activated carbon. The findings highlight that this method offers reduced product losses, enhanced solvent recyclability, and potential economic benefits.

### 4.5 Foam fractionation

**FIGURE 3:**
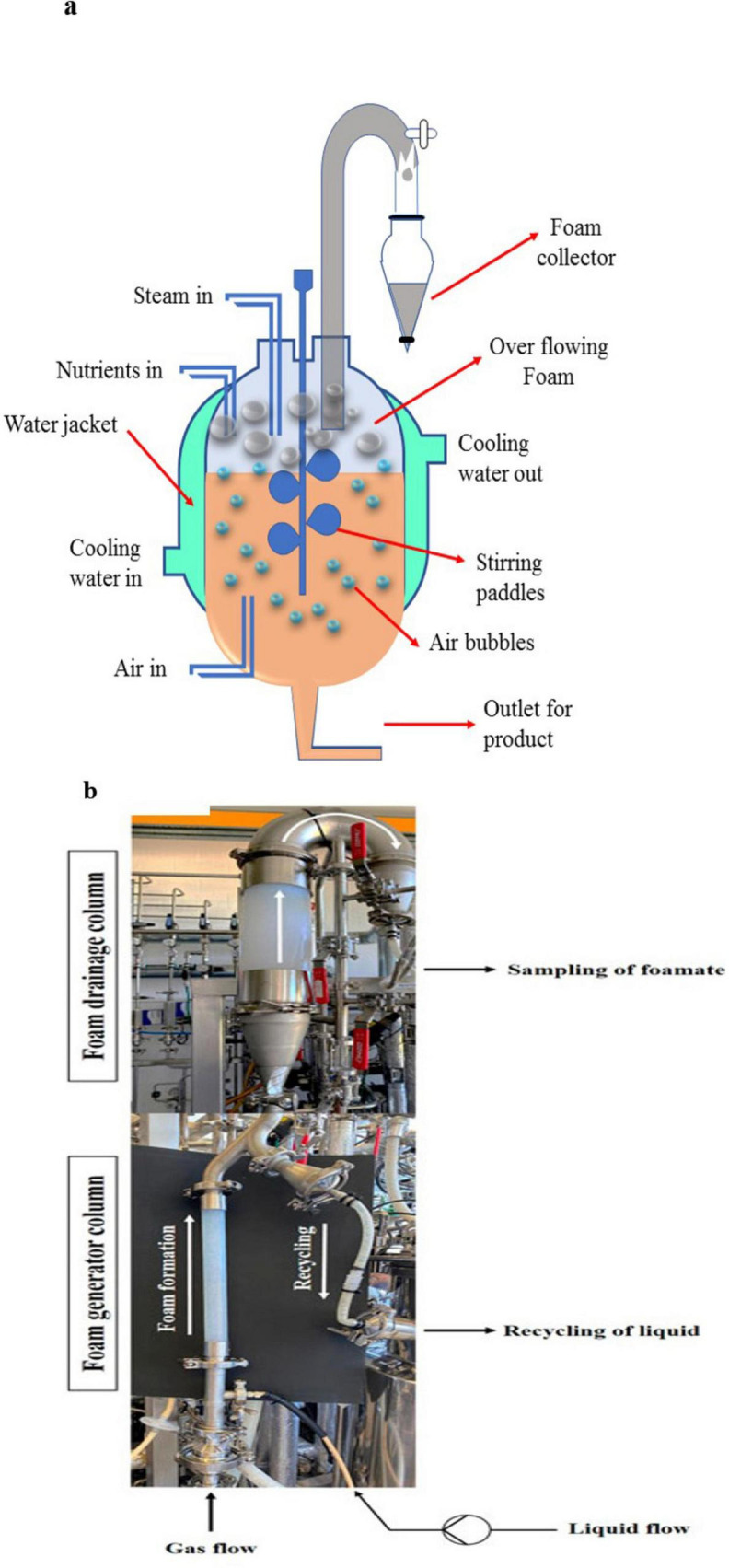
**(a)** A schematic diagram of foam fractionation for recovering yeast-derived emulsifiers. **(b)** This image shows the external foam column, highlighting its design and how it works. The column is connected to the bioreactor. Because of its size, the lower part (foam generator column) and the upper drainage column were photographed separately. The two sections were then merged at their connection point. The white arrows indicate the direction of foam flow and recirculated liquid (Treinen et al., 2023). Reproduced from Treinen et al. (2023).

Foam fractionation is a relatively new method for recovering biosurfactants and emulsifiers. This technique relies on the ability of biosurfactants to associate with the air bubbles, separating them from the liquid medium. Foam fractionation involves generating air bubbles in the fermentation broth, where biosurfactants bind to the bubbles and rise to the surface, forming a foam. The foam is then removed, by connecting the foam to a collecting vessel for example (Winterburn et al., 2011), and the emulsifiers are recovered by further processing e.g., by acidification. A study on foam fractionation for *Pseudomonas tsukubaensis* showed that the technique could recover emulsifiers at a concentration of 1.3 g/L from the culture broth (de Andrade et al., 2017). This method is particularly useful for biosurfactants that have good surface activity, such as glycolipids, lipopeptides, and MELs. Continuous foam fractionation is also a promising method for large-scale applications, where biosurfactant-rich foam is removed continuously from fermentation vessels (Valkenburg et al., 2024). Foam fractionation is cost-effective and requires minimal energy input compared to other extraction techniques. It also allows continuous emulsifier recovery, which can streamline industrial operations. Moreover, the technique does not require organic solvents, making it a promising alternative for the food industry. However, the effectiveness of foam fractionation depends on the foaming properties of the biosurfactant. Some emulsifiers, especially those produced from hydrophobic carbon sources, may not foam effectively, limiting their recovery by this method. Additionally, biomass may be unintentionally removed along with the foam, reducing reactor productivity (challenges in maintaining consistent foam stability and the need for specialized equipment) (Venkataraman et al., 2022; de Fernandes et al., 2023; Valkenburg et al., 2024). Figure 3a shows a foam fraction method for recovering yeast biosurfactants and emulsifiers. One feature of this setup is shown in Figure 3b, where the external foam column operates separately from the bioreactor system and allows recycling of cells and media effectively (Treinen et al., 2023).

### 4.6 Enzymatic extraction

Enzymatic extraction is gaining traction in this sector as a selective and low energy method for extracting yeast emulsifiers, particularly mannoproteins. This method utilizes enzymes to break down the yeast cell wall, releasing the emulsifiers into the surrounding medium. Enzymes such as glucanases, proteases, and mannanases are employed to degrade specific components of the yeast cell wall (Qiao et al., 2022; Zeko-Pivac̆ et al., 2023). For example, zymolyase, which contains β-1,3-glucanase, is commonly used to hydrolyze the yeast cell wall and release mannoproteins with high purity (Qiao et al., 2022). This method is particularly effective for extracting mannoproteins with high mannan-to-protein ratios, which are essential for emulsification properties. Enzymatic treatment of *Saccharomyces cerevisiae* with zymolyase yielded mannoproteins that showed superior emulsifying capacity compared to those extracted via chemical methods (Li and Karboune, 2018). Enzymatic extraction is highly selective and preserves the functional structures of the emulsifiers, such as the mannose-to-protein ratio, which is critical for emulsifying performance. Additionally, enzymatic methods are mild and avoid the use of harsh chemicals. Nevertheless, the primary drawback of enzymatic extraction is its cost. Moreover, enzymatic treatments can be time-consuming, and the process may require optimization to avoid co-extraction of undesirable components. Enzyme recycling strategies, such as covalent binding to nanoparticles, are being explored to lower costs and improve process efficiency (Timira et al., 2024). Varelas et al. (2016) described a β-glucan extraction method based on endogenous enzymatic self-disruption of yeast cells (autolysis) followed by acid extraction, highlighting its potential as a sustainable and environmentally friendly approach. Enzymatic methods are often combined with mechanical techniques to improve emulsifier yields. For instance, combining enzymatic treatment with ultrasonication or bead milling enhances the disruption of yeast cells and increases emulsifier recovery.

### 4.7 Challenges in microbial biosurfactant production

Scaling up microbial biosurfactant production to an industrial level presents several challenges, particularly from an economic perspective, as microbial surfactants are currently more expensive to produce than synthetic surfactants (Santos et al., 2016; Vuc̆urović et al., 2024). This is due to several factors, such as the complex extraction and purification processes involved in microbial surfactant production, which account for nearly 60% of the total production costs (Rizvi and Verma, 2024). For example, using solvents such as chloroform, which are not environmentally benign, to extract microbial surfactants adds extra costs, as these solvents must be removed during the purification step completely for the food sector (Santos et al., 2016). Additionally, the productivity and yield of microbial surfactants, particularly from some wild-type yeast strains, are relatively low (Zaparoli et al., 2020).

However, scientists are working on strain improvement, metabolic engineering, and bioprocess optimization to enhance bioproduction yields. For example, an increased yield of extracellular biosurfactants with enhanced emulsifying activity (1.39 UE d^–1^) was achieved in a *Saccharomyces cerevisiae* strain compared to the control (0.54 UE d^–1^). This improvement was reportedly induced by ultraviolet radiation, which altered the metabolic pathway through stress mediation (Zaparoli et al., 2020). Rhamnolipid biosurfactants are naturally produced by the pathogenic microorganism *Pseudomonas aeruginosa* (Henkel et al., 2017). However, a study demonstrated that recombinant *Saccharomyces cerevisiae* showed significantly higher lipid accumulation within lipid bodies compared to the control, facilitating rhamnolipid biosurfactant production (Bahia et al., 2018). The engineered *S. cerevisiae* strains produced mono-rhamnolipids from sucrose. In this study six enzymes from *P. aeruginosa* involved in mono-rhamnolipid biosynthesis were expressed in the strains. Furthermore, its SUC2 invertase gene was disrupted and a sucrose phosphorylase gene from *Pelomonas saccharophila* was also expressed to reduce the pathway’s overall energy requirement. This study showed the potential to further improve rhamnolipids production in a yeast-based industrial bioprocess (Bahia et al., 2018).

Addressing challenges in microbial biosurfactant production is essential, particularly for target markets such as the food industry (Santos et al., 2016). While microbial biosurfactant production is feasible on a small scale, scaling up remains challenging due to the high costs associated with separation techniques like column chromatography, which are not cost-effective for large-scale applications (Santos et al., 2016). A promising strategy to reduce production costs is the utilization of byproducts and agricultural waste as in-expensive substrates (Santos et al., 2016; Vuc̆urović et al., 2024). Using inexpensive materials such as oily frying residues, whey waste, and potato processing residues not only lower production expenses but also promote sustainable biosurfactant production (Santos et al., 2016; Vuc̆urović et al., 2024). Additionally, a significant challenge limiting the adoption of biosurfactants in the food industry is the lack of comprehensive toxicity data, which remains a critical barrier to wider application (Vuc̆urović et al., 2024).

## 5 Applications of yeast-derived emulsifiers in the food industry

Common emulsions, such as water-in-oil (w/o) and oil-in-water (o/w) types, along with more complex forms such as water-in-oil-in-water (w/o/w) and oil-in-water-in-oil (o/w/o), are essential components in a variety of food products, including butter, yogurt, cheese, meat products, as well as aerated “whipped” items, to name but a few (McClements and Jafari, 2018; Thraeib et al., 2022). Bio-emulsifiers are high-molecular-weight compounds with emulsion-stabilizing properties, produced by various microorganisms under both normal and extreme environmental conditions (Monteiro et al., 2010). For example, *Rhodotorula*, a yeast genus commonly found in polar and glacial habitats, includes several species known for producing large amounts of sophorolipid biosurfactants (Schultz and Rosado, 2020). Bio-emulsifiers derived from *Saccharomyces cerevisiae*, has been successfully used in the food industry to stabilize oil-in-water emulsions (Monteiro et al., 2010; Moreira et al., 2016). Beyond their positive effects on human health such as enhancing the bioavailability of fat-soluble vitamins, bio-emulsifiers are increasingly valued for their potential as natural ingredients in food. This includes a growing interest in yeast-derived emulsifiers as versatile, health-promoting additives (Thraeib et al., 2022). The following discusses various yeast-derived components which have been directly as emulsifiers in food applications.

### 5.1 Mannoproteins

Yeast mannoproteins, amphiphilic in nature, have excellent emulsifying and stabilizing properties. These molecules consist of carbohydrate portions that ensure solubility and protein portions that provide the emulsifying ability. Their use is particularly prevalent in emulsified food products like salad dressings, mayonnaise, and meat products. Mannoproteins from *Saccharomyces uvarum* have shown to be effective in emulsifying and stabilizing oil-in-water emulsions in mayonnaise, replacing egg yolk and synthetic stabilizers such as xanthan gum without affecting sensory qualities. Mayonnaise formulations containing 0.6/100 g, 0.8/100 g, and 1.0/100 g of mannoproteins exhibited increased stability over 28 days, and demonstrated higher stability compared to formulations prepared with xanthan gum. Sensory evaluation scores for aroma, color, flavor, and overall evaluation were similar for both formulations (mannoproteins or xanthan gum), with no significant preference indicated by the evaluation panel regarding purchase intent (da Silva Araújo et al., 2014).

A French salad dressing formulated with mannoprotein from brewer’s spent yeast (*Saccharomyces uvarum*) showed improved stability and sensory acceptance over 28 days compared to soy lecithin-based formulations (de Melo et al., 2015). An improved texture, consistency, and sensory properties of salad dressings were also reported when a carbohydrate–lipid–protein complex bioemulsifier from *Candida utilis* was used in their preparation. In formulations, concentrations ranging from 0.2% to 0.8% of the bioemulsifier were employed, with 0.7% proving optimal for texture improvement (Campos et al., 2019).

Mannoproteins have also been effectively used in meat emulsions such as sausages. For example, the mannoprotein MP112 extracted from baker’s yeast biomass by enzymatic hydrolysis using β-1,6-glucanase GluM showed an excellent emulsifying properties were used to reduce the fat content of sausages while increasing moisture and protein levels (Zhong et al., 2023). Replacing pork fat with MP112 emulsions at a ratio of 50%–75% led to improved textural properties, including higher hardness, chewiness, and cohesiveness. This formulation also resulted in a healthier fatty acid profile with a higher polyunsaturated fatty acid/saturated fatty acid ratio and a lower n-6/n-3 ratio. Additionally, the oxidative stability of the sausages was significantly improved. In applications involving meat protein gels, mannoproteins like MP112 enhanced gel strength and water-holding capacity (Zhong et al., 2023). When added to porcine myofibrillar protein solutions at concentrations of 10%–20% (v/v), MP112 significantly improved the gel’s ability to immobilize water. This made it a suitable fat alternative in low-fat meat products, improving not only the texture but also the nutritional quality of the end product (Qiao et al., 2022).

Mannoproteins from *Saccharomyces cerevisiae* KA01 have been used in processed foods as a natural emulsifier. The emulsion stability of various vegetable oils, including soybean oil, palm oil, corn oil, olive oil, sunflower oil, rice bran oil, and sesame oil, prepared with mannoprotein extract, was similar to that of emulsions prepared with gum Arabic or lecithin. Specifically, the emulsification activity for emulsions containing mannoproteins was 57%–64.7%, for those containing gum Arabic, 58%–65.8%, and for those containing lecithin, 54.3%–64.2%. Furthermore, palm oil-in-water emulsions prepared with mannoproteins extract exhibited high emulsification activity across a broad pH range (5–8) and in the presence of up to 3% (w/v) sodium chloride and up to 0.1% (w/v) CaCl_2_ and MgCl_2_ in the aqueous phase. Temperature did not affect the emulsification activity of the mannoproteins extract (Dikit et al., 2010).

### 5.2 Brewer’s spent yeast

Brewer’s spent yeast (BSY), a by-product of the beer industry, is a sustainable source of mannoproteins, polysaccharides and other emulsifying agents to be effective in stabilizing emulsions in various food applications. BSY extracts have been tested in oil-in-water emulsions, showing excellent stabilizing properties. These extracts, rich in proteins and mannoproteins, were used to replace egg yolk and starch in mayonnaise formulations (Reis et al., 2023). In one study, BSY extracts were employed at concentrations as low as 1%, yielding emulsions that were comparable in texture and stability to commercial emulsifiers (Dikit et al., 2010; Li et al., 2020). BSY extracts obtained through alkaline extraction were used to replace modified starch (E1422) in mayonnaise formulation, at one-third of the usual concentration (Reis et al., 2023). This approach not only provided stable emulsions but also allowed for the development of vegan and clean-label alternatives for traditional mayonnaise, without compromising texture or stability.

### 5.3 Applications of other yeast-derived emulsifiers

Other biosurfactants produced by yeast species, such as *Candida utilis* and *Saccharomyces cerevisiae*, have exhibited excellent emulsifying, stabilizing, antimicrobial, and antioxidative capabilities, making them ideal for various food applications. For example, salad dressings containing biosurfactants from *Saccharomyces cerevisiae* and commercial emulsifiers like Tween 80 and xanthan gum displayed enhanced texture, and their emulsifying activity remained stable at pH 7 and 9 (Ribeiro et al., 2022). These formulations were able to emulsify oils such as corn, sunflower, and palm oil with an emulsification index above 50%. The biosurfactant concentrations ranged from 0.2% to 0.8%, with 0.7% being optimal for achieving the desired texture (Ribeiro et al., 2022).

Biosurfactants such as lipopeptides derived from different yeast species have been successfully used in bakery products to improve dough handling, texture, and product quality. For example, a glycolipid biosurfactant produced by *Saccharomyces cerevisiae* URM 6670 was used to replace egg yolk in a cookie formulation. The biosurfactant exhibited high heat resistance, with negligible mass loss at temperatures up to 200 °C. Incorporating the biosurfactant into the cookie dough did not alter the cookie’s physical or physicochemical properties after baking. Furthermore, analysis of the texture profile before baking revealed that substituting egg yolk with the biosurfactant did not change firmness, cohesiveness, or elasticity compared to the standard formulation (Ribeiro et al., 2020).

Rhamnolipids have also been employed in dairy products such as ice cream, yogurt, and buttercream. For example, rhamnolipids were shown to improve the texture and extend the shelf life of these products by reducing lipid oxidation (Debnath et al., 2022). The biosurfactant Mannosylerythritol lipids-A (MELs-A), produced by *Pseudozyma aphides*, has recently been shown to improve dough quality when added at concentrations of 0.5%, 1.0%, and 1.5%. Increasing MEL-A concentrations from 0% to 1.5% correlated with increasing G’ and G” values, indicating that MEL-A significantly enhanced the rheological properties of frozen dough. Differential scanning calorimetry (DSC) analysis revealed that 1.5% MEL-A reduced freezable water by 1.88%, suggesting that higher MEL-A concentrations decrease water fluidity and enhanced water hydration within the dough. Further analysis demonstrated that MEL-A modified gluten proteins and strengthened the gluten network, as evidenced by molecular weight distribution and microstructure observations, effectively protecting the dough structure from ice crystal damage. Final product evaluations showed that MEL-A improved loaf volume, gas retention, and bread texture. Notably, MEL-A exhibited strong antibacterial activity against vegetative cells and spores of *Bacillus cereus*, with limited inactivation of yeasts. In a simulated dough fermentation, 1.5% MEL-A resulted in a 99.97% reduction in vegetative cells and a 75.54% reduction in spores of *Bacillus cereus* (Shu et al., 2022). Table 3 shows examples of yeast-derived emulsifiers in the food industry with their functions.

**TABLE 3:** Yeast-derived emulsifiers in the food industry.

Yeast-derived emulsifiers	Food application	Function	References
Mannoproteins from *Saccharomyces uvarum*	-Replacing egg yolk and synthetic stabilizers in mayonnaise	-Emulsifying and stabilizing oil-in-water emulsions - Not affecting sensory qualities	da Silva Araújo et al., 2014
Mannoproteins from brewer’s spent yeast *Saccharomyces uvarum*	-French salad dressing	-Improved stability and sensory acceptance over 28 days	de Melo et al., 2015
Carbohydrate–lipid–protein complex bioemulsifier from *Candida utilis*	-Salad dressings	-Improved texture, consistency, and sensory properties	Campos et al., 2019
Mannoproteins MP112	-Meat emulsions -Sausages -Replacing pork fat -Meat protein gels -Fat alternative in low-fat meat products	-Reduce the fat content of sausages -Increasing moisture and protein levels - Improved textural properties -Higher hardness, chewiness, and cohesiveness -Healthier fatty acid profile -Higher polyunsaturated fatty acid/ saturated fatty acid ratio -Lower n-6/n-3 ratio -Improved the oxidative stability -Enhanced gel strength and water-holding capacity - Improved the gel’s ability - Improving nutritional quality	Qiao et al., 2022; Zhong et al., 2023
BSY extracts	- Replace egg yolk in mayonnaise -Replace modified starch (E1422) in mayonnaise	-Excellent stabilizing properties - Stable emulsions -Development of vegan and clean-label alternatives for traditional mayonnaise	Reis et al., 2023
Biosurfactants from *Saccharomyces cerevisiae*	-Salad dressings	-Enhanced texture -Stable emulsifying activity at pH 7 and 9	Ribeiro et al., 2022
Lipopeptides biosurfactants	-Bakery products -Dough formulations -Replace egg yolk in cookie -Cupcakes	-Improve dough handling, texture, and product quality -Better gas retention and texture enhancement -Dough maintaining similar physicochemical properties -Optimal dough quality	Kiran et al., 2017; Ribeiro et al., 2020
Rhamnolipids biosurfactants	-Dairy products -Ice cream -Yogurt -Buttercream -Buttercream pastries	-Improve the texture -Extend the shelf life -Improved emulsification -Creamier texture -Enhanced aeration properties	Azevedo et al., 2024; Muthusamy et al., 2008

Yeast-derived biosurfactants and proteins have also been explored for their potential in advanced emulsion technologies such as nanotechnology and Pickering emulsions. Rhamnolipids, have been used to develop lipid-based nanosystems for food applications. For example, oil-in-water nanoemulsions (NEs) with droplet sizes smaller than 150 nm were created using low-energy methods with rhamnolipids, which showed high surface activity and similar interfacial properties to Quillaja saponin, a commercial natural surfactant (Bai and McClements, 2016). These nanoemulsions offer enhanced stability and can be used for the encapsulation and delivery of bioactive compounds in food systems (Azevedo et al., 2024).

Yeast protein particles have also been employed to stabilize Pickering emulsions, which are emulsions stabilized by solid particles instead of surfactants. Yeast proteins, when subjected to treatments like sonication, form particles that stabilize oil-in-water emulsions through a Pickering mechanism. These proteins adsorb onto the oil-water interface, forming a strong interfacial layer that prevents droplet coalescence. In high internal phase Pickering emulsions (HIPPEs), yeast protein particles were used to stabilize oil droplets, with optimal sonication conditions yielding particles of smaller size and higher amphiphilicity (Cheng et al., 2024).

The emulsification index of yeast biosurfactants can reach up to 61.2% under extreme conditions such as high temperatures (120 °C) (Petra de Oliveira Barros et al., 2024). In salad dressing formulations, yeast-derived biosurfactants were used to stabilize emulsions with an emulsification activity of 50%–60% for oils like corn, sunflower, and palm oil (Ribeiro et al., 2022). Concentration and usage rates of yeast-based emulsifiers vary depending on the type of the emulsifier and the food product. Biosurfactants i.e., a lipopeptide derived from *Nesterenkonia* sp. in bakery and cookie dough formulations were applied at concentrations ranging from 0.75% to 2%, with 0.75% being sufficient to replace egg yolks (Kiran et al., 2017). In mayonnaise, BSY mannoproteins were used at concentrations of 1% to replace traditional emulsifiers like egg yolk and modified starch (Reis et al., 2023).

## 6 Conclusion and future perspective

Yeasts are a promising route to produce a range of natural emulsifiers, offering diverse applications within the food industry. Yeast-derived emulsifiers, sourced from over 20 species, encompassing whole-cell biomass, cell wall fractions, mannoproteins, β-glucans, and exopolysaccharides, present exceptional emulsifying and stabilizing capabilities. These natural alternatives not only enhance the textural, stability, and sensory attributes of food products but also provide a sustainable substitute for synthetic emulsifiers, addressing growing consumer concerns regarding artificial additives. Successful incorporation of yeast-based emulsifiers into various food matrices, including mayonnaise, dairy products, meat emulsions, and bakery items, underscores their significant potential for widespread industrial application. Compared to traditional synthetic emulsifiers, yeast-derived biosurfactants offer several key advantages. Their inherent biodegradability and lower toxicity align with the increasing demand for clean-label and eco-friendly food production. Furthermore, they can be produced from renewable resources, contributing to a more sustainable bioeconomy. In certain applications, yeast-derived emulsifiers exhibit superior functionality, such as enhanced emulsion stability under specific pH or temperature conditions, or improved interactions with other food components. This positions this class of compounds, not just as more sustainable replacements, but as potentially superior alternatives to existing emulsification solutions.

Several key trends are driving the increased interest and production of yeast-derived biosurfactants. The rising consumer awareness of the health impacts of synthetic food additives is a major factor. Consumers are actively seeking natural, recognizable ingredients, pushing the food industry to explore bio-based alternatives. Simultaneously, the focus on sustainable and environmentally friendly production processes is growing, favoring biosurfactants produced through microbial fermentation over chemically synthesized compounds. Advances in biotechnology and bioprocessing are also playing a crucial role, enabling more efficient and cost-effective production of yeast emulsifiers.

Despite the considerable promise of yeast-derived emulsifiers, several challenges must be addressed to facilitate their broader adoption. Efficient and cost-effective extraction and purification methods remain a critical area for optimization. Current processes can be complex and yield relatively low quantities of the desired emulsifiers, increasing production costs. Further research is needed to develop scalable and sustainable extraction techniques that minimize environmental impact. Advancements in biotechnology, including strain engineering, metabolic pathway optimization, and fermentation process control, hold the key to enhancing production efficiency and yield. Tailoring yeast strains to overproduce specific emulsifiers or optimizing fermentation conditions to maximize productivity are promising strategies. Furthermore, a deeper understanding of the molecular mechanisms governing the emulsifying properties of yeast-derived compounds is essential. Elucidating the structure-function relationships will enable the design of more effective and targeted applications across diverse food systems. Future research should prioritize scaling up production while ensuring consistent functionality and quality. This will involve optimizing bioreactor design, fermentation parameters, and downstream processing. Developing novel food formulations that leverage the unique properties of yeast-based emulsifiers, potentially in combination with other bio-based stabilizers, could unlock new possibilities for clean-label, functional, and texturally innovative food products. Addressing regulatory hurdles and conducting comprehensive safety assessments are also crucial steps for gaining widespread acceptance and regulatory approval within the food industry.

In conclusion, the ongoing shift toward natural, sustainable, and health-conscious food choices positions yeast-derived emulsifiers as a viable and sustainable alternative to conventional synthetic emulsifiers. Continued advancements in fermentation technology, biotechnology, and food formulation, coupled with rigorous safety evaluations, will pave the way for yeast-based emulsifiers to play an increasingly significant role in shaping the future of food emulsification and the development of next-generation food products.
